# *In situ*, direct observation of seasonal embolism dynamics in Aleppo pine trees growing on the dry edge of their distribution

**DOI:** 10.1111/nph.18208

**Published:** 2022-06-01

**Authors:** Yael Wagner, Feng Feng, Dan Yakir, Tamir Klein, Uri Hochberg

**Affiliations:** ^1^ Plant & Environmental Sciences Department Weizmann Institute of Science Rehovot 7610001 Israel; ^2^ Institute of Soil, Water and Environmental Sciences Volcani Center ARO Rishon Lezion 7505101 Israel; ^3^ Earth and Planetary Science Department Weizmann Institute of Science Rehovot 7610001 Israel

**Keywords:** embolism visualization, optical visualization, *Pinus halepensis* (Aleppo pine), seasonal embolism dynamics, μCT

## Abstract

- Xylem embolism impairs hydraulic conductivity in trees and drives drought‐induced mortality. While embolism has been monitored *in vivo* in potted plants, and research has revealed evidence of embolism in field‐grown trees, continuous *in situ* monitoring of cavitation in forests is lacking.
- Seasonal patterns of embolism were monitored in branchlets of Aleppo pine (*Pinus halepensis*) trees growing in a dry Mediterranean forest. Optical visualization (OV) sensors were installed on terminal branches, in addition to monthly sampling for micro computed tomography scans.
- We detected 208 cavitation events among four trees, which represented an embolism increase from zero to *c*. 12% along the dry season. Virtually all the cavitation events occurred during daytime hours, with 77% occurring between 10:00 and 17:00 h. The probability for cavitation in a given hour increased as vapor pressure deficit (VPD) increased, up to a probability of 42% for cavitation when VPD > 5 kPa.
- The findings uniquely reveal the instantaneous environmental conditions that lead to cavitation. The increased likelihood of cavitation events under high VPD in water‐stressed pines is the first empirical support for this long hypothesized relationship. Our observations suggest that low levels of embolism are common in Aleppo pine trees at the dry edge of their distribution.

## Introduction

Failure of the water transport system due to cavitation is thought to be a major mechanism of drought‐induced tree mortality (Tyree & Zimmermann, 2002; Barigah *et al*., 2013). This happens when the tension in the xylem conduits increases, either due to low water availability, increased evaporative demand or an interaction between the two. When the tension exceeds a critical threshold, an air bubble is sucked through the pit membrane into a functional conduit, expands and blocks it (i.e. ‘embolism’ or ‘cavitation’; Zimmermann, 1983). Due to the rapid dynamics of the process and the metastable state of the water in the conduits, quantifying embolism *in situ* has posed a great methodological obstacle to scientists. Several studies have quantified embolism in field‐grown trees, relying on indirect measurements. For example, in some cases, a species‐specific vulnerability curve (VC) was taken at one time point, and then used to quantify native embolism in the field, relying on Ψ_x_ measurements (e.g. Koepke & Kolb, 2013; Martin‐StPaul *et al*., 2014). Such practice ignores the potential plasticity of the VC (Awad *et al*., 2010; Plavcová *et al*., 2011; Sorek *et al*., 2021), as well as inherent variation between plants or even organs (Johnson *et al*., 2022) that could potentially tip the scales from cavitation to no cavitation; that is, a seasonal change or intraspecific variation in the susceptibility to cavitation can lead to erroneous estimation of the cavitation based on instantaneous Ψ_x_ measurements. In other studies, native embolism was quantified in field‐grown trees by estimating changes in hydraulic conductivity of stems or branches (Trifilò *et al*., 2015; Klein *et al*., 2016; Love & Sperry, 2018). However, both outer‐xylary sources of hydraulic resistance (Scoffoni *et al*., 2017) and artefacts related to the sampling protocol (Wheeler *et al*., 2013; Torres‐Ruiz *et al*., 2015) may affect the final absolute values. Therefore, while these studies have played an important role in advancing our understanding of xylem embolism dynamics and mechanisms, our knowledge is constrained by the fact that a cavitation event was never observed in a forest‐grown tree, *in situ*. As a result, testing the relationship between cavitation and instantaneous environmental conditions is impossible. Common perceptions, such as the link between high vapor pressure deficit (VPD) and cavitation (e.g. Schultz & Matthews, 1997; Breshears *et al*., 2013; Cochard *et al*., 2021; Mantova *et al*., 2021), are essentially theoretical, and even the assumption that cavitation occurs at midday (Tyree & Zimmermann, 2002; Choat *et al*., 2012; Trifilò *et al*., 2017) has never been directly tested.

The new optical visualization (OV) method (Brodribb *et al*., 2016) allows a unique opportunity to measure cavitation in intact plants (Johnson *et al*., 2022). It consists of the continuous imaging of the plant’s xylem and relies on the fast change in light reflectance at the moment of cavitation (Brodribb *et al*., 2016). The method had proved suitable for a variety of plants (e.g. Brodribb *et al*., 2017; Hochberg *et al*., 2019; Gauthey *et al*., 2020; Johnson *et al*., 2022) and could thus provide the missing information on cavitation in the forest. As a reference technology, the micro computed tomography (μCT) method has become increasingly common in plant hydraulics for the quantification of embolism *in vivo* (Cochard *et al*., 2015). This technique relies on the sample’s transmittance to X‐ray in order to distinguish water from air‐filled conduits. The OV and μCT techniques were found to produce similar results in both angiosperms and gymnosperms (Gauthey *et al*., 2020; Johnson *et al*., 2020) and complement each other well, as the OV method allows for continuous, *in situ* and *in vivo* quantification of cavitation events, while μCT allows direct observation of embolism levels in the entire xylem section. We used these two visualization techniques to directly quantify embolism in *Pinus halepensis* trees growing in Yatir forest, at the dry edge of their distribution. *Pinus halepensis* is a widely spread species around the Mediterranean (Atzmon *et al*., 2004; Klein *et al*., 2011, 2013), yet ongoing drying and warming have put its southern distribution at risk (Patsiou *et al*., 2020). This makes it a highly relevant and interesting case study for the occurrence of embolism in field‐grown trees. We hypothesized that during the dry season, when the low soil water content (SWC) exerts a significant limitation on the trees’ ability to rehydrate, embolism develops during heat waves.

## Materials and Methods

### Site description and experimental design

The Yatir forest is an afforestation site near the northern edge of the Negev desert, Israel (31°20′N, 35°03′E). It is populated mainly with *P. halepensis* trees, planted in the 1960s at their ecological range edge (Klein *et al*., 2011). The climate is dry Mediterranean, with mean annual precipitation of 280 mm that occurs exclusively during the winter (November–March). The summer is long (May–October) and dry, with no rain events. Mean diurnal temperatures and VPD range from *c*. 10°C and < 1 kPa (respectively) in January to 25°C and 2–3 kPa (respectively) in July, with short heat waves during the spring (April–May) and autumn (September–October), when midday temperatures may climb to over 40°C and VPD spikes to as high as 6 kPa (Tatarinov *et al*., 2016). These values, specifically those common for the summer and heat waves, represent extremely high atmospheric demand compared to other habitats (Turner *et al*., 2012; Klein *et al*., 2014).

During the spring of 2021, four plots were chosen in the Yatir forest, in a well‐studied stand (e.g. Grünzweig *et al*., 2003; Rotenberg & Yakir, 2010). Three to four trees of similar age (*c*. 56 yr) with a height of 7–13 m and a diameter at breast height (DBH) of 10–30 cm were marked in each plot. The trees were monitored for embolism (see next sections) and for midday xylem water potential (Ψ_x_) once or twice per month from the end of the rainy season and throughout the summer (April–October). For Ψ_x_ measurements, terminal branchlets were covered with sealed aluminum foil bags for at least 30 min before their sampling, at which point they were immediately measured with a Scholander‐type pressure chamber (Scholander *et al*., 1965; PMS Instrument Company, Albany, OR, USA). In addition, sensors for the optical visualization of cavitation were installed on four trees and were monitored continuously throughout the study period (see next section). Soil water content was monitored continuously using Trime PICO‐32 sensors (IMKO Micromodultechnik GmbH, Ettlingen, Germany) that were installed in three pits in one of the plots, at depths of 5, 15, 30 and 50 cm. The data were averaged over the four measured depths and then over the different pits, in order to represent SWC of the stand. Temperature and relative humidity were monitored continuously above the canopy at the meteorological and flux tower located within the stand, and were used to calculate VPD.

### Embolism quantification – optical visualization sensors

To obtain a general view of embolism susceptibility of the trees, five shoots were harvested in June 2021 (when embolism was < 3%) and transferred to the lab for measurements of VC using the OV method (see Supporting Information Methods S1 for a detailed description of the VC procedure).

For direct *in situ* observation of the cavitation dynamics, in one of the plots four trees were fitted with OV sensors. One branchlet with an average diameter of 0.5–0.6 cm from each tree was selected for the OV measurement during the period of May–September 2021. Monitored branchlets were of sunlit branches at a height of 1–2 m. A small section (rectangle of 1.5 × 0.5 cm) of the bark and phloem from each selected branchlet was carefully removed, followed by a thin coverage of conductive adhesive gel (Aquasonic Clear; Parker Laboratories Inc., Fairfield, NJ, USA) to improve light transmission and reduce heterogeneity in the speed of desiccation across different xylem layers.

**Fig. 1:**
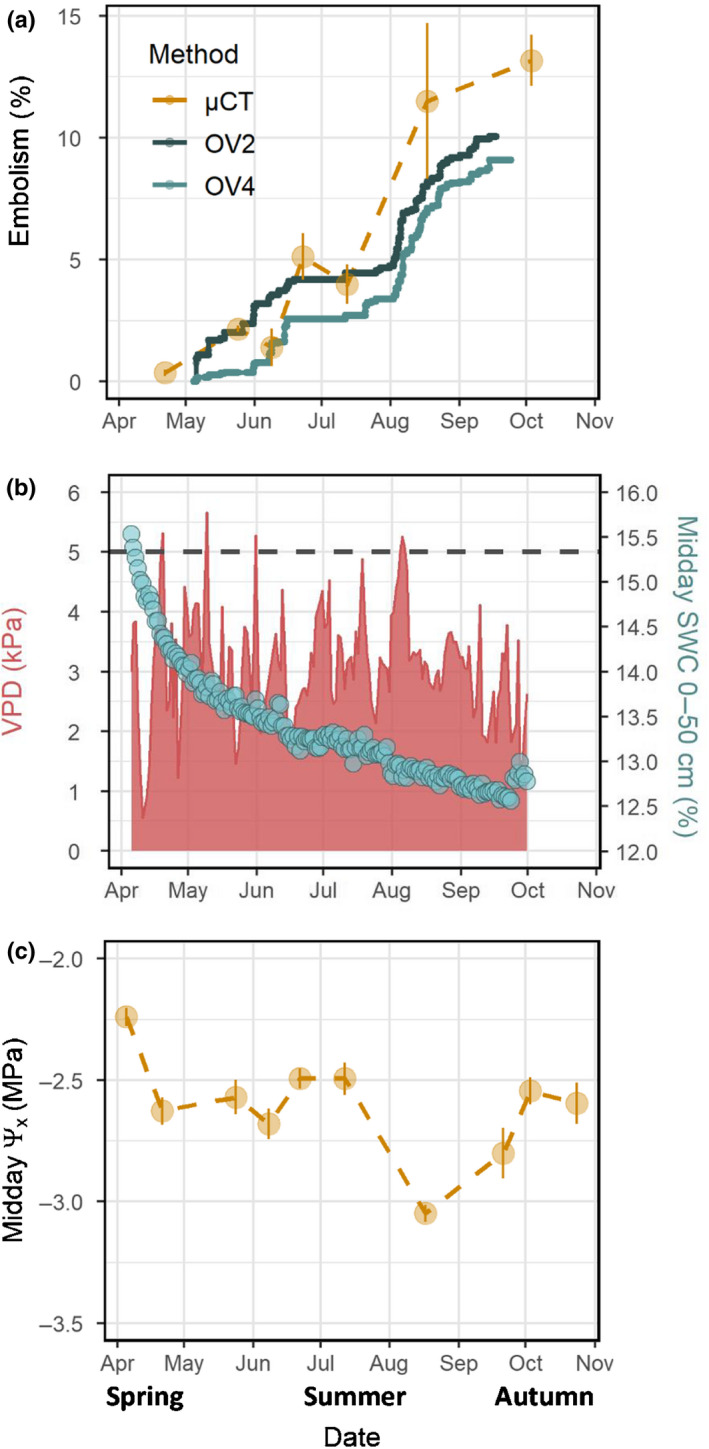
(a) Seasonal dynamics of embolism in mature *Pinus halepensis* trees, quantified as percentage cavitated pixels using the optical visualization (OV) method, or as percentage loss of conductive area using the μCT method. Bars represent standard error (*n* = 4). (b) Seasonal dynamics of vapor pressure deficit (VPD; red) and soil water content (SWC; blue). Dashed line indicates a threshold of VPD = 5 kPa. (c) Seasonal dynamics of Ψ_x_. Bars represent standard error (*n* = 4).

A custom‐built clamp was used to fix the exposed xylem in place, in which a camera was installed right over the xylem. A Raspberry Pi microcomputer was connected to control and stored images produced by the camera every 5 min. The imaged area was magnified by a ×20 lens and illuminated by an LED light source that provided reflected light from the xylem surface. The abrupt increase in light reflection during continuous imaging was interpreted as xylem embolism (Brodribb *et al*., 2016). After installation, the clamp was covered with adhesive tape to isolate the imaged area from external heat and the setup was connected to the Israeli national power grid. Due to our limited ability to spatially resolve cavitation of individual tracheids, the increase in embolism between two consecutive images was considered as one cavitation event. The OV method can also capture embolism refilling (Hochberg *et al*., 2017; Johnson *et al*., 2018). Accordingly, we also analyzed the images to detect abrupt decreases in light reflection that could be interpreted as xylem refilling. The measurements were initiated during early May, when Ψ_x_ was −2.6 MPa (Fig. 1c) and terminated in early October, 10 d following the first rain. The branchlets used for OV measurements were then collected and transferred to the lab with the sensors in place for further dehydration. Imaging was restarted immediately upon arriving at the lab (3 h in total with no imaging). Image analysis before and after transportation to the lab ensured that no cavitation had taken place during that time. In the lab, the branchlets were air‐dehydrated until fully embolized. Complete embolism was assumed following 24 h with no cavitation. The total embolized pixels were estimated by adding the cavitated pixels that occurred in the forest to those that occurred in the lab. Embolism degree at a given time was expressed as follows:

(Eqn 1) $$\%\;\text{embolism}=100\times \frac{P_{\text{cav}}}{P_{\text{max}}}$$

where % embolism is the percentage embolized pixels (see Fig. 1a), *P*_cav_ is the cumulative cavitated pixels at each time point and *P*_max_ is the maximum cavitated pixels. Details of the optical system, image capturing, post‐image processing, and data analysis, as well as an overview of the OV method, are present at www.opensourceov.org.

**Fig. 2:**
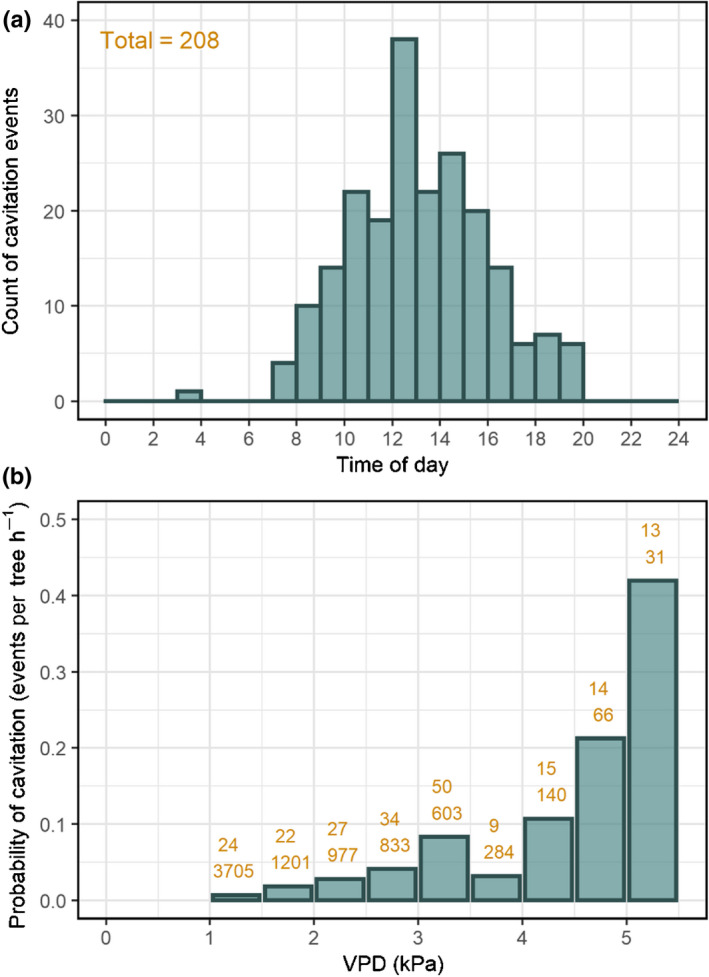
(a) Distribution of cavitation events during the day in mature *Pinus halepensis* trees. (b) Probability of a cavitation event along a vapor pressure deficit (VPD) gradient. The numbers above each column represent the number of cavitation events detected by all four sensors (top row) and the number of hours in that bin (bottom row). Note that the number of VPD hours were summed individually for each sensor, for the periods during which it worked. Therefore, the total number of hours is higher than the length of the season. *n* = 4.

Among the four sensors, two worked continuously throughout the dry season, and were therefore used to quantify the seasonal embolism dynamics relative to the fully embolized area at the end of the season. Technical failures of the other two sensors prevented continuous data‐logging, and they were therefore only used for counting of cavitation events with respect to the instantaneous environmental conditions (see Fig. 2), and not for quantification of embolism dynamics. To avoid possible bias due to uneven monitoring of the different hours of the day, we ensured that the hours with the highest and the lowest representation were at < 5% distance from the average monitoring time. The normalized histogram, which shows the proportion of cavitation in each round hour of the day, appears in Fig. S1.

### Embolism quantification – µCT

The anatomical structure of coniferous xylem, specifically *P. halepensis*, with its relatively short tracheids (i.e. < 2 mm; David‐Schwartz *et al*., 2016), allows the quantification of embolism using cut samples without introducing artificial embolism (Wheeler *et al*., 2013). On seven campaign days during the study period, one terminal, sunlit branchlet from each measured tree was collected during midday hours (11:30–15:00 h). Terminal branchlets at a height of 1–2 m were wrapped with a wet paper towel while still intact to minimize transpiration. Then, a wrapped *c*. 15 cm segment was cut from the tree and sealed in a plastic bag. The needles were gently removed, and the branchlets were rapidly dipped in melted wax (< 60°C) and then in ice water to stop water loss. After fixation, the samples were kept in a sealed plastic bag on ice until brought to the lab. An area with current‐year growth was scanned in the µCT device (RX Solutions, Chavanod, France) located in the Weizmann Institute µCT facility. The scans were conducted under a power of 7 W, with a frame rate of 2 images s^−1^, resulting in a voxel size of 5–7 µm. During the 3 October campaign, each sample was cut at the middle of the scanned area following the first scan. Before it was scanned again, the cut end was sealed with parafilm to avoid further desiccation during the long scan, which would cause movement and interfere with the scan quality. This cut scan was used to represent the maximum conductive area, assuming that all conductive tracheids cavitated following the cut. Normalization with the October cut scan values was used to create comparable parameters to those generated by the OV method and to better follow the seasonal evolution of cavitation. Finally, slices from the 3D models were analyzed using imagej software (Schindelin *et al*., 2012), where the bark and pith areas were cropped‐out, and air‐filled spaces were quantified just for the xylem tissue. Embolism degree was calculated as:

(Eqn 2) $${\text{PLA}}_{\mu \text{CT}}=100\times \frac{E_{\text{native}}}{E_{\text{max‐oct}}}$$

where PLA_μCT_ is the percentage loss of conductive area, *E*_native_ is the embolized area quantified in the first scan of the branchlets and *E*_max‐oct_ is the embolized area quantified in the second scan (after cutting) of the October campaign.

### Statistical analysis

All statistical analyses were done using r software (R Core Team, 2021; RStudio Team, 2021). For the diurnal distribution of cavitation events, the time‐points when events occurred were pooled into hourly bins, and the events within each bin were counted. For the probability of cavitation along a VPD gradient, hourly average VPD values (measured every 30 min) were pooled into bins of 0.5 kPa, counting them for each sensor’s operating periods individually. As a result, the same hour could be counted several times (as many as four times, during periods when all the sensors were functional), and the total number of VPD hours was longer than the study period. The number of cavitation events within each VPD bin was summed for each sensor. Then, the number of events pooled over all four trees was divided by the pooled number of hours within that same bin. This was done to account for the disproportionately low number of hours with high VPD (i.e. > 4 kPa), compared to the abundant number of hours with VPD of 2.5–3 kPa.

## Results

### Seasonal patterns of stress and embolism formation

Our results showed a seasonal increase in embolism levels from 0% at the end of the rain season (April) to 9–10% or 13% (in the OV and μCT methods, respectively; Fig. 1a) at the end of summer (October). It is important to mention that the initial levels of embolism cannot be estimated in the OV method, and was assumed as zero, based on the negligible embolism levels found in the μCT scans from April and May. Two separate periods of a sharp increase in embolism could be roughly identified, namely spring (April–June) and late summer (August–October), with a relatively stable plateau in July. The initiation of both periods was coordinated with heatwaves when VPD surpassed 5 kPa (Fig. 1b). This intraseasonal trend was only partly mirrored by the midday Ψ_x_ measurements. Midday Ψ_x_ decreased from −2.2 during early April to −2.6 MPa during late April, when embolism monitoring began, but then remained quite stable from May to July, during the first period at which embolism levels increased (Fig. 1c). Midday Ψ_x_ decreased again to −3 MPa during August, just after a late summer heat wave, coinciding with the second period of intense cavitation. Throughout the days or along the season, and regardless of Ψ_x_, we did not detect any refilling events (Fig. 1a).

### Diurnal patterns of embolism formation

An advantage of the continuous data obtained via the OV sensors was the ability to pinpoint the time of day when cavitation typically occurred. This analysis demonstrated that practically all cavitation events occurred during the day, with 161 out of the 208 (77%) events that were captured among all four OV sensors occurring between 10:00 and 17:00 h, that is during Ψ_min_ hours (Fig. 2a; Preisler *et al*., 2021). This distribution is probably a function of the crucial effect of VPD on cavitation, which can be seen in Fig. 2b. Specifically, 13 cavitation events occurred during 31 h with VPD > 5 kPa (42%), while only 107 events occurred during a total of 6716 h with VPD < 3 kPa (1.6%). It is important to mention that our data suggest that VPD acted independently of SWC (Fig. S2).

## Discussion

‘If a cavitation event occurred in the forest and no one was around to measure it…’

To date, the actual occurrence of cavitation in field‐grown trees has not been directly observed. In this study, we show, for the first time, direct observations of the seasonal increase in embolism in adult trees in the forest.

### Low levels of cavitation

The low percentages of cavitation detected in field‐grown trees of Aleppo pine (*c*. 12%) are in line with the measured VC (Fig. S3) and with previous μCT‐established VCs for bench dehydrated branches of that species (Oliveras *et al*., 2003; David‐Schwartz *et al*., 2016). These results present a significant discrepancy from a previous study, which found large diurnal changes in embolism levels in these same trees (Klein *et al*., 2016). We suspect that this discrepancy stems from methodological artefacts associated with the measuring protocol of Klein *et al*. (2016). Placing the samples at 95°C immediately after excision to avoid resin secretion, flushing the samples with high pressure (0.5 MPa; which could induce aspiration of the tori), or the very low hydraulic conductivities of the flushed samples could have all contributed to biased conclusions. The combination of our two imaging methods provides reliable evidence for the low embolism values in the measured trees. As the 2021 midday Ψ_x_ dynamics were similar to values previously measured at the same plot (Preisler *et al*., 2022), and because 2021 was not an extremely dry year (Fig. S4), we suspect that the extent and seasonal trend of embolism shown here are common for these trees.

Interestingly some cavitation events did occur early in the season, under low VPD and relatively high SWC (Figs 2, S2). This suggests that within the xylem tissue, some conduits have a higher susceptibility to embolism. These could be protoxylem or defected conduits. These early failing conduits seem disadvantageous or even be regarded as experimental artefacts (Domec & Gartner, 2001; Choat *et al*., 2010). However, their existence is supported by virtually every embolism measurement of plants that were never exposed to any stress (e.g. Hochberg *et al*., 2016). Zero percent embolism is rarely the case. Specifically for pines, 3.5–8% embolism was measured using μCT in well‐watered potted trees (Bouche *et al*., 2016; Rehschuh *et al*., 2020). Since evolution is a tight accountant, we should keep an open mind regarding the potential functional advantages that low cavitation levels confer.

The fact that no single refilling event occurred during the whole experiment is supported by recent studies which failed to observe any xylem refilling in pines even after complete soil rehydration (Hammond *et al*., 2019; Rehschuh *et al*., 2020). At the same time, these studies showed that pines could sustain high levels of cavitation, recovering their conductivity with the formation of new xylem. For example, 50% of *Pinus taeda* trees recovered from 80% embolism (Hammond *et al*., 2019). Along these lines, we believe that the seasonal conductive area loss we show here is fairly low and compensable. Since the canopy area of these mature trees (> 50 yr) may have reached its maximal size, they need to maintain a relatively constant conductive area. It is more than likely that upon soil rehydration, the addition of a new growth ring will add sufficient xylem to compensate for the 12% cavitated conduits.

### VPD‐driven cavitation

Cavitation is thought to be driven by either high VPD and/or low SWC via a decrease in Ψ_x_ below the air‐seeding threshold (Zimmermann, 1983; Cochard, 2019). However, to the best of our knowledge, until now, there has been no robust, data‐based support for that assumption, primarily due to a lack of direct, continuous monitoring of embolism. Interestingly, we found no significant correlation between diurnal SWC and diurnal embolism increment (Fig. S2). We suspect that under drier years (when SWC approaches the tensions that lead to cavitation), a correlation between soil dehydration and cavitation events could be expected. However, under average years (such as 2021; Fig. S4), as long as there is sufficient water in the soil, tight stomatal regulation restricts Ψ_x_ to the embolism avoidance zone (Sperry *et al*., 1998). In support of this, several studies have shown that the Yatir pines had significantly reduced their transpiration during the summer months and maintained a fairly stable Ψ_x_ of *c*. −3 MPa (Grünzweig *et al*., 2003; Kadmiel *et al*., 2008; Raz‐Yaseef *et al*., 2010; Preisler *et al*., 2022). Ψ_x_ reportedly moves from that homeostasis into the cavitation zone only when high VPD enforces high transpiration despite stomatal down regulation (Cochard *et al*., 2021). This idea is supported by our measurements, which showed a significantly enhanced likelihood for cavitation under high VPD. Also, the significant decrease in Ψ_x_ values in August, which followed a heatwave with 7 d of VPD > 4 kPa, highlights the effect of VPD on Ψ_x_. Due to the limited temporal resolution of our Ψ_x_ measurements, and the fact that only midday values were obtained, it is impossible to tell if the high VPD pushed Ψ_x_ values past the −3 MPa homeostasis. Thus, our observations represent a partial view of the relationship between environmental stress factors (i.e. high VPD, low SWC), Ψ_x_ and embolism formation. However, reverse engineering of the *P. halepensis* xylem vulnerability curve (David‐Schwartz *et al*., 2016; Fig. S3) suggests that our data have probably not captured the most extreme values of Ψ_x_. The importance of VPD in driving cavitation is also manifested by the only large drought‐mortality event in the last decade (late 2015 to 2016; Preisler *et al*., 2021), which followed a summer with an exceptionally high number of extreme VPD events (Fig. S4). A high frequency of VPD might even have an enhanced effect on cavitation due to the constrictions it imposes on the tree’s ability to equilibrate with the soil during the night (Preisler *et al*., 2022). Consequently, an increase in the frequency and duration of heatwaves (IPCC, 2019) might exacerbate fatal cavitation levels.

## Competing interests

The authors declare no conflict of interests in preparing this paper.

## Author contributions

YW and FF collected and analyzed the data. YW, TK and UH designed the experiment. DY provided all environmental measurements. YW wrote the manuscript with contributions from all authors. YW and FF contributed equally to this work.

## Supporting information


**Fig. S1** Probability of a cavitation event normalized to the total number of loggings for each round hour.
**Fig. S2** Diurnal increment of percentage embolized pixels, as a function of vapor pressure deficit and soil water content.
**Fig. S3** Vulnerability curve of mature Aleppo pine trees from Yatir forest.
**Fig. S4** Historical climatic matrix for Yatir forest.
**Methods S1** Detailed description of the vulnerability curve procedure.
Please note: Wiley Blackwell are not responsible for the content or functionality of any Supporting Information supplied by the authors. Any queries (other than missing material) should be directed to the *New Phytologist* Central Office.

## Data Availability

The data that support the findings of this study are available from the corresponding author upon reasonable request.
