# Supplementary material for: In situ, direct observation of seasonal embolism dynamics in Aleppo pine trees growing on the dry edge of their distribution
Source: New Phytol. 2022 Jun 1;235(4):1344–50. doi: 10.1111/nph.18208 (PMC9541785; doi:10.1111/nph.18208)
Supplement: Supplementary file 1 — Fig. S1 Probability of a cavitation event normalized to the total number of loggings for each round hour. Fig. S2 Diurnal increment of percentage embolized pixels, as a function of vapor pressure deficit and soil water content. Fig. S3 Vulnerability curve of mature Aleppo pine trees from Yatir forest. Fig. S4 Historical climatic matrix for Yatir forest. Methods S1 Detailed description of the vulnerability curve procedure. Please note: Wiley Blackwell are not responsible for the content or functionality of any Supporting Information supplied by the authors. Any queries (other than missing material) should be directed to the New Phytologist Central Office. [file NPH-235-1344-s001.pdf]

New Phytologist Supporting Information

In situ, direct observation of seasonal embolism dynamics in Aleppo pine trees growing on the dry edge of their distribution

Yael Wagner, Feng Feng, Dan Yakir, Tamir Klein, Uri Hochberg

Acceptance date: 30 April 2022

Methods S1

Vulnerability curve – OV sensors

Five shoots at one time point of June 2021, 80 to 90 cm in length, were excised from the sun-exposed side of the trees, enclosed in a big black plastic bag to avoid water loss, and transferred to the lab. The basal ends (by ca. 10 cm in total) were recut under water in the lab, and the shoots were allowed to rehydrate overnight in buckets filled with tap water while wrapped in a black plastic bag before the dehydration commenced. A small section (rectangle of 1.5×0.5 cm) of the bark and phloem from each collected sample was carefully removed. The exposed section was covered with a conductive adhesive gel (Aquasonic Clear, Parker Laboratories Inc., Fairfield, NJ, USA) in order to improve light transmission and reduce heterogeneity in the speed of desiccation across different xylem layers. The camera clamp assembly was positioned to best view the prepared xylem and fixed in place. Images were taken every 5 min, Ψ_x was measured 2-3 times a day using the Scholander pressure chamber (Model 1505D, PMS, Albany, OR, USA). A best-fit regression model of the Ψ_x versus time was used to determine the Ψ_x of all the images.

The dehydration lasted six days until cavitation events had seized (concluded after 24 hours with no cavitation events). The image sequences were processed using ImageJ, as described in Hochberg *et al.* (2017). The degree of cavitation (in %) was expressed as the cumulative number of cavitated pixels at a specific time normalized to the total number of cumulative pixels under full dehydration (Brodribb *et al.*, 2016). These data were used to determine the VCs, which is the percentage of cavitated area as a function of Ψ_x.

References

Brodribb TJ, Skelton RP, McAdam SAM, Bienaimé D, Lucani CJ, Marmottant P. **2016**.

Visual quantification of embolism reveals leaf vulnerability to hydraulic failure. *New Phytologist* **209**: 1403–1409.
Hochberg U, Windt CW, Ponomarenko A, Zhang Y-J, Gersony J, Rockwell FE, Holbrook NM. **2017**. Stomatal closure, basal leaf embolism and shedding protect the hydraulic integrity of grape stems. *Plant Physiology* **174**: 764–775.

Supporting figures:

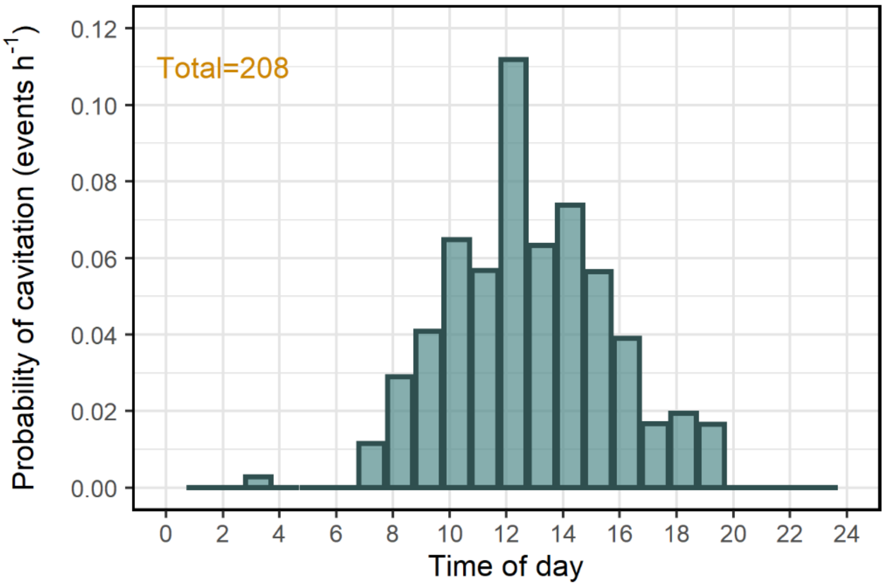

Fig. S1. Probability of a cavitation event in mature *Pinus halepensis* trees. The number of events at a specific hour of the day was normalized to the total number of loggings for each round hour.

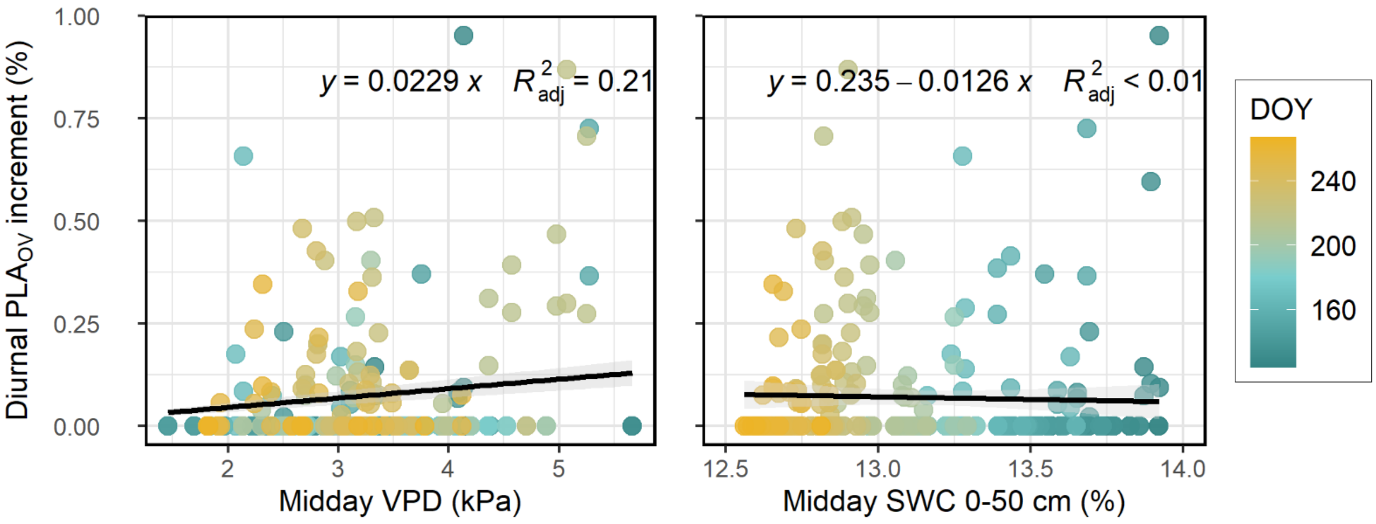

Fig. S2. Diurnal increment of % embolised pixels in *Pinus halepensis*, as a function of midday values of vapor pressure deficit (VPD; A) and soil water content (SWC; B), averaged over the depths of 0-50 cm. Dots' colors represent the day of the Gregorian year (DOY). Black lines represent the linear correlation, and grey shadings represent confidence intervals. *P-value* < 0.001 and =0.6 for VPD and SWC, respectively.

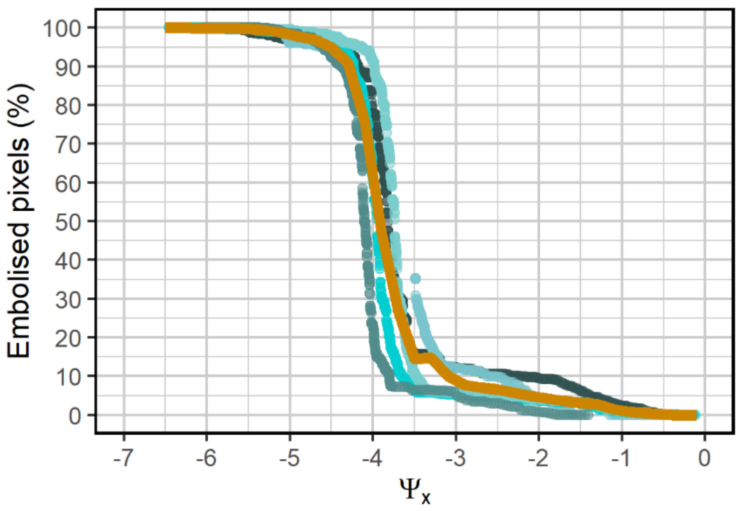

Fig. S3. Vulnerability curve of mature *Pinus halepensis* trees. Different turquoise dots represent the different trees; The orange line represents the mean (*n*=5).

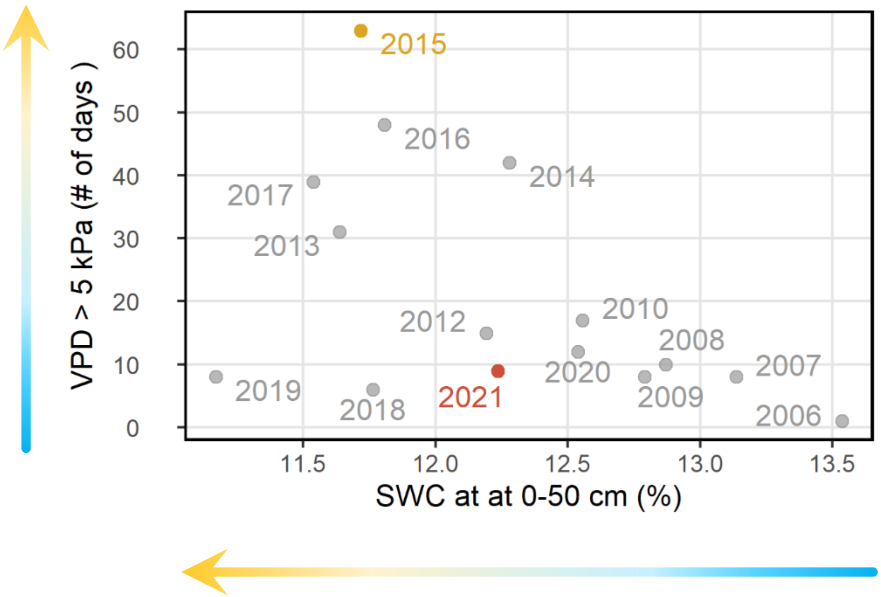

Fig. S4. Climatic matrix in Yatir, mapping the years 2006-2021 (2011 was removed since data was missing for most of the summer), according to their annual minimum soil water content (SWC) averaged over the depths 0-50 cm (%) and the number of days at which vapor pressure deficit (VPD) surpassed 5 kPa. Red and yellow dots mark (respectively) the study period and an extreme year following which extensive tree mortality was observed. Arrows show the direction of air- and soil-dehydration.
